# 

**Published:** 1908-03-14

**Authors:** 


### CONTENTS.

| | PAGE |
|---|---|
| **The Children's Bill** | 617 |
| **Sex and Character** | 618 |
| **Annotations—** | |
| Medical Superintendent's Salaries—The Mental Effects of Influenza—"Pot Still and Patent Still" | 619 |
| **Medical Opinion and Movement** | 620 |
| **Hospital Clinics—** | |
| Intestinal Worms in Children and their Treatment. By Robert Hutchison, M.D., F.R.C.P. | 623 |
| A Demonstration of Selected Skin Cases. By G. Norman Meachen, M.D., B.S.Lond., M.R.C.P. | 625 |
| **Medicine—** | |
| Spinal Hemiplegia—II. | 627 |
| Veronal Rashes | 628 |
| **Points in Surgery—** | |
| The Spontaneous Disappearance of Malignant Growths | 629 |
| Movable Kidney—II. | 630 |
| **Gynæcology—** | |
| Uterine Polypi—I. | 631 |
| **Otology—** | |
| Operations for Otorrhœa | 632 |
| **Therapeutics—** | |
| Prescriptions and Dispensing | 633 |
| **Diseases of Children—** | |
| Influenza in Babies | 634 |
| **Points in Pathology—** | |
| Secretin | 635 |
| **Medico-Legal Points—** | |
| Damages for Libel | 636 |
| **Hospital Administration—** | |
| The Royal Free Hospital | 637 |
| The Vagaries of the Drug Market | 638 |
| News and Coming Events | 640 |
| **Nursing Administration—** | |
| Economy in Nursing Department | 639 |
| **Editor's Letter-Box** | |
| The Hospital Chapel—Still Birth Certificates | 641 |
| **The Graduates' Medical Schools** | 642 |

